# Availability and Quality of Size Estimations of Female Sex Workers, Men Who Have Sex with Men, People Who Inject Drugs and Transgender Women in Low- and Middle-Income Countries

**DOI:** 10.1371/journal.pone.0155150

**Published:** 2016-05-10

**Authors:** Keith Sabin, Jinkou Zhao, Jesus Maria Garcia Calleja, Yaou Sheng, Sonia Arias Garcia, Annette Reinisch, Ryuichi Komatsu

**Affiliations:** 1 Strategic Information and Evaluation Department, the Joint United Nations Programme on HIV/AIDS, Geneva, Switzerland; 2 Technical Advice and Partnerships Department, The Global Fund to fight AIDS, Tuberculosis and Malaria, Geneva, Switzerland; 3 Jiangsu Provincial Center for Disease Control and Prevention, Nanjing, China; 4 Department of HIV/AIDS, World Health Organization, Geneva, Switzerland; 5 Technical Evaluation Reference Group Support Team, The Global Fund to fight AIDS, Tuberculosis and Malaria, Geneva, Switzerland; National HIV and Retrovirology Laboratories, CANADA

## Abstract

**Objective:**

To assess the availability and quality of population size estimations of female sex workers (FSW), men who have sex with men (MSM), people who inject drug (PWID) and transgender women.

**Methods:**

Size estimation data since 2010 were retrieved from global reporting databases, Global Fund grant application documents, and the peer-reviewed and grey literature. Overall quality and availability were assessed against a defined set of criteria, including estimation methods, geographic coverage, and extrapolation approaches. Estimates were compositely categorized into ‘nationally adequate’, ‘nationally inadequate but locally adequate’, ‘documented but inadequate methods’, ‘undocumented or untimely’ and ‘no data.’

**Findings:**

Of 140 countries assessed, 41 did not report any estimates since 2010. Among 99 countries with at least one estimate, 38 were categorized as having nationally adequate estimates and 30 as having nationally inadequate but locally adequate estimates. Multiplier, capture-recapture, census and enumeration, and programmatic mapping were the most commonly used methods. Most countries relied on only one estimate for a given population while about half of all reports included national estimates. A variety of approaches were applied to extrapolate from sites-level numbers to national estimates in two-thirds of countries.

**Conclusions:**

Size estimates for FSW, MSM, PWID and transgender women are increasingly available but quality varies widely. The different approaches present challenges for data use in design, implementation and evaluation of programs for these populations in half of the countries assessed. Guidance should be further developed to recommend: a) applying multiple estimation methods; b) estimating size for a minimum number of sites; and, c) documenting extrapolation approaches.

## Introduction

Globally, the HIV epidemic affects key populations, such as female sex workers (FSW), men who had sex with men (MSM), people who inject drugs (PWID) and transgender women, disproportionately [1–5]. Legal and social barriers can prevent key population members from seeking health-related and social services. These barriers, upheld by stigma and discrimination, and systemic indifference or discomfort to discuss the behaviors that both define these populations epidemiologically and sociologically, combine to drive these populations underground. UNAIDS estimated that PWID, MSM, sex workers had 28, 19 and 12 times higher HIV prevalence levels than that among adults in the general population in countries which reported prevalence data for both the general population and key populations. This pattern appears not only in countries with concentrated epidemics but also in countries with generalized epidemics [5]. To be successful, public health interventions for a robust HIV response require monitoring key population members’ interactions with prevention, care and treatment services. A strong response, that will halt the HIV epidemic among key populations, must reach at least 80% of key population members with services [6].

The use of services among key populations, which is low across different groups in many countries [5]. Only a handful of Asian and sub Saharan African countries have national-scale programs for sex workers [5]. A review of 54 FSW-oriented projects found the delivered services are often condom distribution only, occasionally offering HIV testing [6]. A 2011 review concluded that less than 10% of gay and other men who have sex with men receive a basic package of HIV prevention interventions [7] while in 2014, UNAIDS found a declining trend in service coverage from 59% in 2009 to 40% in 2013 based on data reported from 20 countries [5]. PWID who were reached by needle and syringe programs over the previous 12 months in 85 countries ranged from 20% to 60%, while opioid substitution therapy coverage across 79 countries ranged from 20% to 40% [5].

To plan, monitor and evaluate programs designed to mitigate the impact of the HIV epidemic on these key populations, reasonable estimates of the population sizes are necessary. Size estimates have greatest programmatic value when used locally but there remains an important need for national figures for planning and resource allocation [8–10].

The size of key populations can be estimated using different methods, each having strengths and weaknesses [11]. Multiple methods are recommended to generate an estimate at a specific site for a given group [12]. Extrapolations from a single or a few sites to generate a national estimate can be a simple applications of the proportion of estimated group members obtained from the site (s) with estimation exercises to the total adult population applied to the remaining geographic areas, or a complex approach by accounting for other factors: socio-economic factors, geographic area, and different subgroups [13–15].

United Nations and donor agencies have held numerous training workshops in many countries to expand and improve population size estimates of each of the four aforementioned key populations, as well as piloted new approaches [16]. Methods to develop population size estimates are described in UNAIDS/WHO guidelines [2]. The present paper reviews the quantity and quality of recent population size estimates and provides recommendations for further improvements to such estimates. Some reports included here have not been officially published; therefore, this paper does not provide actual estimates of key populations by countries, while the official data are publicly available at www.aidsinforonline.org.

## Methods

### Data sources

The following data sources were examined: Country Progress Reports for the United Nations General Assembly Special Session on AIDS (UNGASS) and Global AIDS Response Progress Report (GARPR) to UNAIDS [17], AIDS Data Hub and gray literature reports [18], annexes to Global Fund concept notes and programmatic gap analysis tables [19], and publicly available documents including integrated bio-behavioral surveillance (IBBS) reports, population size estimation (PSE) reports, journal articles available on PubMed, grey literature available on websites, and technical meeting abstracts. The assessment was limited to 140 countries which were eligible for the Global Fund grants in 2010 (see Appendix). Despite broad search of peer-reviewed and grey literature, this assessment relied primarily on reports submitted by national HIV/AIDS programs to UNAIDS or donor agencies since few published size estimates were endorsed by national authority and therefore are not used for national programming.

### Categorization

Size estimates were only considered if they were generated since 2010. Categories were developed to reflect the utility of the estimates to HIV programmes. A full assessment of the underlying data quality was beyond the scope of this analysis. They were categorized, with the availability and quality in descending order from (a) to (e,) according to criteria that considered compositely: estimation methods, geographic coverage, extrapolation approached and key population groups covered. In each domain, a determination was made as to whether empirical data could be used for national planning, local planning, or did not exist. Data with no documented methods could be used for planning but would be considered of lesser value than empirical estimates.

1. **Nationally adequate**: estimates are empirically derived using one of the following methods: 1) multiplier; 2) capture-recapture; 3) mapping/enumeration; 4) network scale up method (NSUM) or population-based survey; 5) respondent driven sampling–successive sampling (RDS-SS). Estimates had to be national or a combination of multiple sites with a clear approach to extrapolating to a national estimate; and at least two major population groups of national interest are included.
2. **Nationally inadequate but locally adequate in selected sites**: estimates are empirically derived using 1) multiplier; 2) capture-recapture; 3) mapping; 4) NSUM or population-based survey; 5) RDS-SS. Estimates are only from sites where targeted programs are available but are insufficient for national program use. Estimates are available for at least two major population groups of national interest.
3. **Documented estimates but inadequate methods**: estimates are derived from 1) expert opinions; 2) Delphi; 3) wisdom of crowds; 4) programmatic results or registry or 5) regional benchmarks. Estimates may or may not be national;
4. **Undocumented or untimely**: estimates are reported but not documented or were derived prior to 2010.
5. **No data**: no size estimates are reported.

Geographic coverage was assessed using the following criteria, bearing in mind that a) and b) give national or close to national estimates, while c-e provide local estimates:

1. **National sample** designed to generate a national estimate, or estimates are reported as national.
2. **More than 50% of subnational divisions**: 50% or more of the first order subnational divisions are covered by estimates with both urban and rural sites included.
3. **Less than 50% of subnational divisions**: less than 50% of the first order subnational divisions are covered with both urban and rural sites included.
4. **Major cities**: the exercise was done in selected major cities with no rural areas covered.
5. **Capital city**: estimate only from the capital.

The extrapolation approach is classified by strength of approach toward yielding a national estimate.

1. **Proportion of adult males or females**: national estimates are calculated based on a proportion or a range of proportions of adult males or females, who are key population community members.
2. **Summed up**: national estimate is the sum of site-specific estimates with no adjustment.
3. **Regression or probability formula**: regression models were used to estimate populations in areas without an estimation exercise using information from those areas where estimations are available.
4. **Based on one selected number**: national estimate is extrapolated based on one estimate among a number of estimated numbers.
5. **Delphi or consensus**: a formal process considering different factors to arrive at an estimate.
6. **No extrapolation**.

The number of different estimates for a site was based on the actual number of estimates provided in the report, irrespective of methods used. For example, the number of estimates will be 2 if two multipliers were used.

Maps were generated using Q-GIS (version 2.8, http://www.qgis.org/en/site/). UNAIDS regional divisions were used, while country specific shape files were downloaded from Global Administrative Areas (http://www.gadm.org/country).

## Results

### Availability

Among 140 low and middle income countries assessed for the availability of population size estimates, 99 have at least one estimate for one population since 2010, 87 countries had 2 or more groups, 54 had for 3 or 4, 8 had estimates for all 4 populations. By population, 87 countries had estimates for FSW, 88 countries for MSM, 53 countries for PWID and 17 for transgender women (Table 1).

**Table 1:** Number of countries with available estimates for female sex workers (FSW), men who have sex with men (MSM), people who inject drug (PWID) and transgender women by groups over the year 2010–2014 in low- and middle-income countries.

Groups	2010	2011	2012	2013	2014
FSW	8	8	17	23	31
MSM	9	11	19	23	26
PWID	3	8	12	19	11
Transgender women	1	2	3	5	6

### Categorization

**Fig 1:**
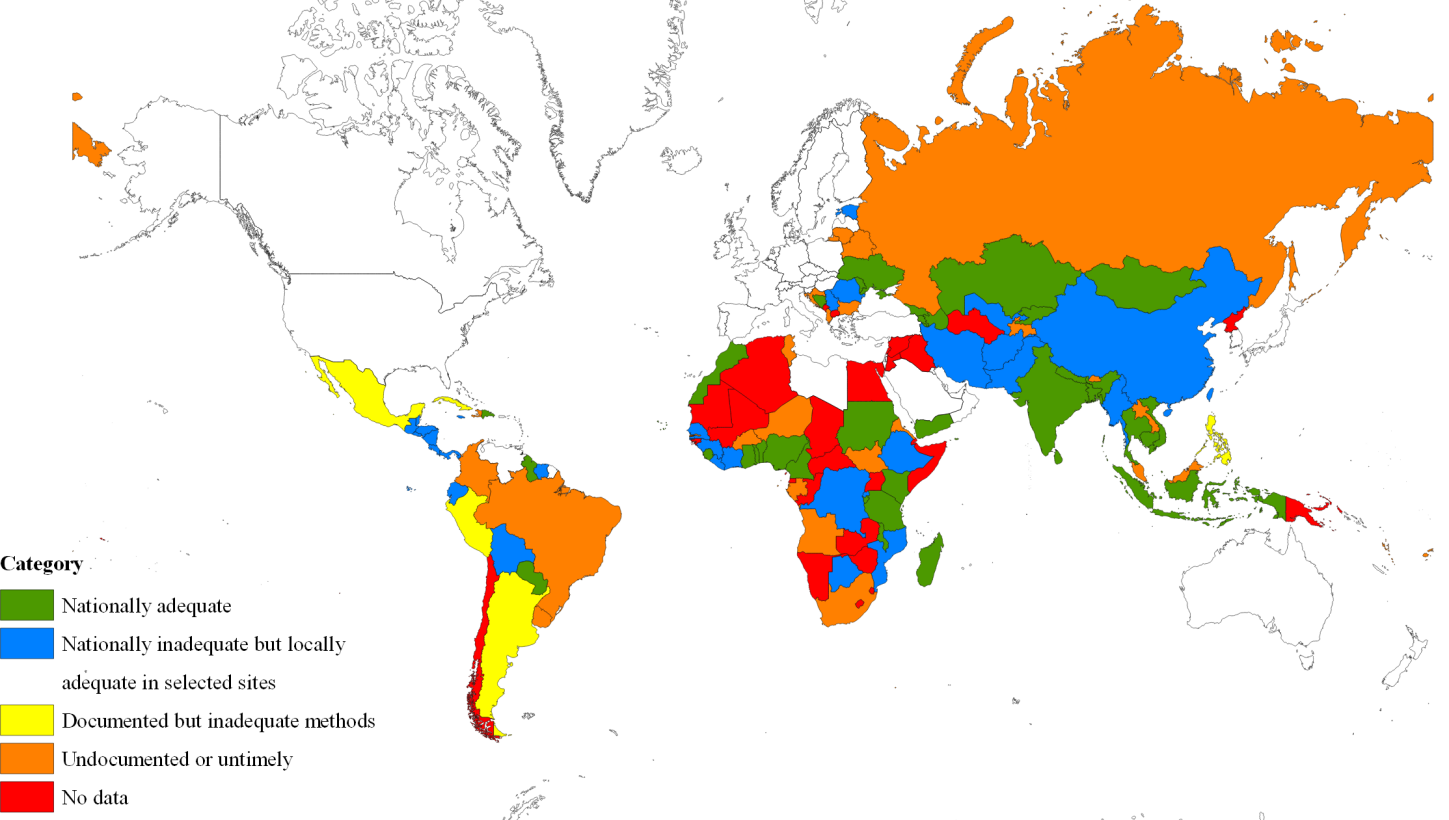
Categorization of population size estimates of female sex workers, men who have sex with men, people who inject drugs, and transgender women in low- and middle-income countries, 2010–2014.

Among 99 countries with at least one estimate, 38 countries were categorized as having nationally adequate estimates, 30 as nationally inadequate but locally adequate in areas where estimation exercises were done, 7 as inadequate documentation of methods for their estimates, 24 having untimely or undocumented estimation data (Fig 1). Country specific information by key population groups can be found in the S1 Table.

### Estimation approaches

Table 2 presents methods used to generate size estimates. Multiplier, capture-recapture (CRC), census and enumeration, and programmatic mapping were the most commonly used approaches. RDS-SS has been applied to generate estimates for all four groups in a few settings. Network Scale-Up Method has been applied in a number of countries for FSW, MSM and PWID.

**Table 2:** Application of methods in estimating population size estimates for female sex workers (FSW), men who have sex with men (MSM), people who inject drug (PWID) and transgender women in low- and middle-income countries, 2010–2014.

Methods	FSW	MSM	PWID	Transgender women
Multiplier	29	31	23	2
Capture re-capture	19	17	11	2
Census & enumeration	19	10	3	1
Programmatic mapping	27	23	12	9
Network scale up method or population-based survey	4	11	9	
RDS-SS successive sampling	2	3	2	
Administrative registry/programmatic results	2	1	1	1
Regional benchmark	3	7	2	1
Population-based survey	1	5	4	
Expert opinion (wisdom of crowds/literature/ Delphi/key informants)	13	10	7	2
Wisdom of crowds	6	10	3	
Not Reported	9	6	6	2
Total number of countries	87	88	53	17

### Number of estimation data points

Most countries (Table 3) had one size estimate for each measured population. Fewer than 20% of countries had 3 or more estimates for the respective populations.

**Table 3:** Number of estimates used in countries with known estimation methods for female sex workers (FSW), men who have sex with men (MSM), people who inject drug (PWID)and transgender women in low- and middle-income countries, 2010–2014.

Number of estimates	FSW	MSM	PWID	Transgender women
Five or more	2	4	5	
Four	4	2		
Three	12	6	7	1
Two	15	16	8	1
One	45	54	27	13
Total number of countries	78	82	47	15

### Geographic coverage

Among countries with reported size estimation methods, almost half covered the entire country based on a national sample or extrapolations. Around 1/5 covered only the capital or major cities (Table 4).

**Table 4:** Geographic coverage of estimation in countries with known estimation methods for female sex workers (FSW), men who have sex with men (MSM), people who inject drug (PWID) and transgender women in low- and middle-income countries, 2010–2014.

Geographic coverage	FSW	MSM	PWID	Transgender women
National or national representative sample	41	43	31	8
More than 50% of first subnational administrative divisions	10	6	3	1
Less than 50% of first subnational administrative divisions	10	7	3	3
Major cities	9	17	7	2
Capital city	6	8		1
Not reported	2	1	3	
Total number of countries	78	82	47	15

### Extrapolation

More than 20% of countries used a proportion of the population of interest among adult men or women to extrapolate the size estimates. More than 1/3 of countries had no extrapolation or did not mention extrapolation methods. Less than 15% of countries used regression models based on multiple demographic and socioeconomic variables (Table 5).

**Table 5:** Approaches used for extrapolations to national population size estimates in countries with known estimation methods for female sex workers (FSW), men who have sex with men (MSM), people who inject drug (PWID) and transgender women in low- and middle-income countries, 2010–2014.

Approaches for extrapolations	FSW	MSM	PWID	Transgender women
Proportion of adult population	21	30	10	4
Based-on one selected estimate	4	2	4	
Summed up from site-specific results	8	7	4	1
Regression or models	9	13	6	4
Delphi/consensus	6	5	5	1
Total number of countries with extrapolations	58	57	29	10

## Discussion

This article presents a global review of population size estimations for four epidemiologically key populations, FSW, MSM, PWID and transgender women, in low- and middle-income countries from 2010 to 2014. More than two-thirds of countries have estimates for at least one of the four populations. More than two thirds of the estimates were empirically derived while more than half of the estimations were extrapolations from a varying number of sites for the entire country or were based on a national sample. Despite a decade of calls for such estimates, only 38 countries are deemed nationally adequate while 30 countries are locally adequate. Together this represents less than 50% of 140 low and middle incomes countries have useful estimates.

Estimates of population proportions (data not shown) were broadly similar to previously published, decade-old estimates [3, 20–22]. This suggests that population proportions of these groups are relatively constant within a limited range over time and across contexts. Variability is likely attributable to different factors for each population. Sex workers often congregate where men without families live. Gay and other men who have sex with men may preferentially migrate to cities where they can live anonymously, out of sight of stigmatizing eyes. PWID are often more numerous closer to drug trafficking routes. However, routes are fungible and shift under pressures from interdiction efforts, potentially spreading injection drug use, HIV and hepatitis C virus to new communities with every shift. Each of these factors, combined with socio-cultural differences, which can be linked to legal differences, contribute to heterogeneity of distribution of these key populations, making population proportions difficult to apply for extrapolation across social and political contexts.

Globally there is no detailed guidance for extrapolation from estimate(s) made at discrete site(s) to determine national numbers. Currently, the proportion of key populations out of adult males or females is widely used. In the process of deriving such a proportion, some countries adjusted based on understanding of differences between site(s) where exercises were done and remaining sites. Some adopted directly the proportion obtained from the exercises at site(s). Some countries used regression models to extrapolate their estimates. In these countries, additional information, related to the key population, were entered into a model. For example, for FSW estimates, degree of urbanization, number of hotels, development of tourism, adult male to female ratio, extent of migration, and business opportunities were collected and used for refining extrapolations [14]. Some countries used one estimate from one method to represent the entire country. A number of other countries went through a Delphi or consensus process to reach an estimate. The varied practices applied may inform the development of detailed guidance for extrapolation, including criteria for extrapolation, such as minimum number of sites with estimates before a national estimate can be derived with a range

Each size estimation method has both strengths and limitations, there is no gold standard for size estimation for key populations. It is important to apply multiple methods to generate multiple estimates to triangulate to reach both a point estimate and a range. This assumes the combined trueness of all estimates. Application of multiple methods can generate a wrong estimate whenever the truth is outside the range of the estimates. Among countries with known estimation methods, more than half generated national estimates based on a single estimate and therefore are making national policy decisions based on very limited data and local decisions in the absence of important data. Less than one fifth of national estimates were based on 3 or more estimates, suggesting that few countries have adequate estimates from which to plan nationally. Among countries with national estimates, only 9 countries provided a point and a range of estimates for FSW, 13 for MSM, 7 for PWID, 4 for transgender women (data not shown). It is possible to have multiple estimates in one estimation process, for example, several multipliers in one IBBS. Multiple estimates may balance the biases among them. Multiple estimates should be triangulated with programmatic data to generate possible boundaries of the estimates [23–25].

Traditional estimation methods, such as multiplier and capture-recapture, remain the most commonly applied in estimating sizes of all four key populations. For FSW and MSM, one-third of estimates used census, enumeration or programmatic mapping. These methods not only estimate the sizes of the populations, but, if employed to their full power, can improve the understanding the typology and places where risk behaviors happen [26–28]. New chatting and dating tools and apps, used with internet or smartphones, present challenges for all approaches, especially mapping-based, as well as new opportunities for prevention service promotion. RDS-successive sampling, despite its application in a limited number of countries, presents a new alternative whenever an RDS survey is the source of data [29].

Most estimates in countries with known methods covered both urban and rural areas. However, few of the reports in these countries provided descriptions of how urban sites compare with rural sites, or urban to rural ratios. Less than a quarter of estimates were built upon the exercises in major cities or capital cities only. While it is fair to assume key populations in cities have more episodes of risky behavior, understanding the size and behavioral patterns of key populations in rural areas is crucial for tailored, efficient response planning and implementation as evidenced by recent rural HIV outbreaks among PWID [30].

Definitions of each of the populations were diverse. Definitions differ by description of sexual behaviors (anal, oral, vaginal) or sex work (exchange sex for money, goods, benefits, convenience, etc.,) or injection (intravenous or intramuscular or subcutaneous or other routes) or drug (heroin, amphetamine-type substances, other drugs, etc.,) and time frame (life time, past 12 months, past 6 months, past 3 months, etc.) These local definitions may accommodate local programming. However, they render comparability across countries and regional or global aggregation more challenging.

This assessment is confined to the data and reports available to UNAIDS, WHO and the Global Fund, and in the published literature. However, we believe the assessment captured the majority of available estimates made between 2010 and 2014 for the four key populations in low- and middle-income countries. There might be some missed reports from estimation exercises but the bias resulted from unfound reports is hard to quantify.

Population size estimates for FSW, MSM, PWID and transgender women are increasingly available but still limited globally, hampering national programs’ abilities to monitor the HIV prevention, care and treatment needs of key populations and national programs’ ability to evaluate their service coverage. Current guidance would benefit from reemphasizing that multiple size estimation methods should be applied whenever possible to generate multiple estimates with uncertainty bounds in a large number of sites with appropriate urban and rural distribution. Technical guidance on extrapolation approaches should be further developed. Guidance for national programs should emphasize that: a) population size estimates primarily help monitor and evaluate local HIV service programs for the respective populations; b) HIV case-based surveillance data should be analyzed to indicate where communities of the populations exist and might benefit from programs, which will subsequently require size estimates; c) key population communities should be engaged to mitigate any risks related to such exercises; and, d)national size estimates are most useful for national planning needs and international donors; while local size estimates are useful to each locality that has or needs adequate HIV prevention or intervention services.

### Appendix

**The list of countries:** Afghanistan, Albania, Algeria, Angola, Argentina, Armenia, Azerbaijan, Bangladesh, Belarus, Belize, Benin, Bhutan, Bolivia, Bosnia and Herzegovina, Botswana, Brazil, Bulgaria, Burkina Faso, Burundi, Cabo Verde, Cambodia, Cameroon, Central Africa Republic, Chad, Chile, China, Columbia, Comoros, Republic of Congo, Democratic Republic of Congo, Costa Rica, Cote d’Ivoire, Croatia, Cuba, Djibouti, Dominica, Dominica Republic, Ecuador, Egypt, El Salvador, Equatorial Guinea, Eritrea, Estonia, Ethiopia, Fiji, Gabon, Gambia, Georgia, Ghana, Grenada, Guatemala, Guinea, Guinea Bissau, Guyana, Haiti, Honduras, India, Indonesia, Iran, Iraq, Jamaica, Jordan, Kazakhstan, Kenya, Kiribati, Democratic People’s Republic of Korea, Kosovo, Kyrgyzstan, Laos, Lebanon, Lesotho, Liberia, Lithuania, Macedonia, Madagascar, Malawi, Malaysia, Maldives, Mali, Marshall Islands, Mauritania, Mauritius, Mexico, Micronesia, Moldova, Mongolia, Montenegro, Morocco, Mozambique, Myanmar, Namibia, Nepal, Nicaragua, Niger, Nigeria, Pakistan, Palestine (West Bank and Gaza), Panama, Papua New Guinea, Paraguay, Peru, Philippines, Romania, Russia Federation, Rwanda, Samoa, Sao Tome and Principe, Senegal, Serbia, Seychelles, Sierra Leone, Solomon Islands, Somalia, South Africa, South Sudan, Sri Lanka, St. Lucia, St. Vincent & Grenadines, Sudan, Suriname, Swaziland, Syria, Tajikistan, Tanzania, Thailand, Timor Leste, Togo, Tonga, Tunisia, Turkmenistan, Tuvalu, Uganda, Ukraine, Uruguay, Uzbekistan, Vanuatu, Viet Nam, Yemen, Zambia, Zimbabwe

## Supporting Information

S1 Table Categorization of availability and quality by key population groups. (XLS)
